# The role of deep learning for periapical lesion detection on panoramic radiographs

**DOI:** 10.1259/dmfr.20230118

**Published:** 2023-10-18

**Authors:** Berrin Çelik, Ertugrul Furkan Savaştaer, Halil Ibrahim Kaya, Mahmut Emin Çelik

**Affiliations:** 1 Oral and Maxillofacial Radiology Department, Faculty of Dentistry, Ankara Yıldırım Beyazıt University, Ankara, Turkey; 2 Electrical Electronics Engineering Department, Faculty of Engineering, Gazi University, Ankara, Turkey; 3 Biomedical Calibration and Research Center, Gazi University Hospital, Gazi University, Ankara, Turkey

**Keywords:** lesion, detection, deep learning, artificial intelligence, dentistry, diagnosis

## Abstract

**Objective:**

This work aimed to detect automatically periapical lesion on panoramic radiographs (PRs) using deep learning.

**Methods:**

454 objects in 357 PRs were anonymized and manually labeled. They are then pre-processed to improve image quality and enhancement purposes. The data were randomly assigned into the training, validation, and test folders with ratios of 0.8, 0.1, and 0.1, respectively. The state-of-art 10 different deep learning-based detection frameworks including various backbones were applied to periapical lesion detection problem. Model performances were evaluated by mean average precision, accuracy, precision, recall, F1 score, precision-recall curves, area under curve and several other Common Objects in Context detection evaluation metrics.

**Results:**

Deep learning-based detection frameworks were generally successful in detecting periapical lesions on PRs. Detection performance, mean average precision, varied between 0.832 and 0.953 while accuracy was between 0.673 and 0.812 for all models. F1 score was between 0.8 and 0.895. RetinaNet performed the best detection performance, similarly Adaptive Training Sample Selection provided F1 score of 0.895 as highest value. Testing with external data supported our findings.

**Conclusion:**

This work showed that deep learning models can reliably detect periapical lesions on PRs. Artificial intelligence-based on deep learning tools are revolutionizing dental healthcare and can help both clinicians and dental healthcare system.

## Introduction

Periapical lesions are one of the most common pathologies and they are defined as boundaries that limit microorganisms’ spread into the neighboring structures.^1^ Clinic and radiographic examination using intra- or extraoral images are fundamental for assessing prevalent clues related to periapical lesion diagnosis. Radiographic evaluation can provide accurate diagnosis. Panoramic radiographs (PRs) are frequently used in dental clinical routines as a standard screening modality for assessment, diagnostic purposes and treatment planning.^2^ It scans a wide range of oral structures with relatively lower radiation dose. Dentists, radiologists and surgeons specialized in oral and maxillofacial dentistry interpret PRs during clinical practices. Previous studies show that the success rate of image reading in the medical field depends on the physician’s training; similarly, in dentistry, the success rate fluctuates depending on knowledge, experience, ability, and biases.^3^ Inconsistency in PRs evaluation process may lead to misdiagnosis followed by mistreatment.^3^ Goldman et al. reported that only a 50% agreement rate was seen among experts for endodontic pathosis evaluation.^4^ Moreover, the same experts confirmed their previous diagnosis less than 85% when they were re-evaluated in several months.^4^ Periapical lesion detection and diagnosis may also be very time-consuming in experts’ daily routines.

Overall, considering the rapidly growing trend of artificial intelligence research and dental imaging modalities, an automatic, reliable and robust decision-support system is significantly needed for clinicians.

Artificial intelligence (AI) has been applied to many specialties in dentistry both to improve the clinical decision-making process and to support disease detection and treatment planning steps.^5–11^ As a subset of AI, deep learning is a machine learning method that teaches computers by example directly from images, text or sound. With the help of a huge amount of labeled data and computing power, deep learning models learn features now from data without needing any handcrafted feature extraction process. While conventional neural networks have around three hidden layers, deep learning models can have up to 150 layers, achieving higher accuracy than before. Consequently, it brings significant attention to deep learning from various research fields in recent years. A wide range of applications covers oral and maxillofacial surgery, cariology and endodontics, dental implantology, periodontics and orthodontics.^11,12^

A decision-support system based on AI is now a need for clinicians. This work aimed to detect periapical lesions using state-of-art deep learning detection models on PRs. The image pre-processing phase followed the data preparation step before delivering the data to the deep learning models. 10 different deep learning models were comparatively used for periapical lesion detection on PRs. Model performances were evaluated and presented by well-known evaluation metrics.

## Methods and material

**Figure 1.**
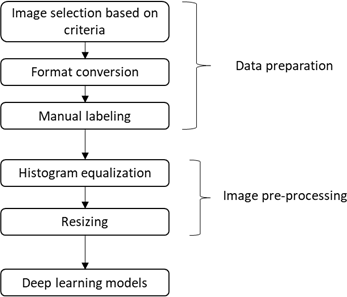
Flowchart of the proposed approach.

This study was performed under the Helsinki Declaration standards and approved by the Ethical Review Board of the University (approval number 2023–79). Figure 1 presents the systematic research design and the methodology employed.

### Data preparation

Data preparation phase includes data selection, conversion of data to the proper format, and data splitting before applying deep learning models. The dental panoramic device, Planmeca oy, Helsinki, Finland, generated the images with 66–70 kVp, 8–12.5 mA, 15.8–16 s parameters. Anonymized PRs were randomly chosen among patients older than 18 years old between 2022 and 2023 from university hospital radiology archive. PRs with metallic artifacts, position-based distortions, and incomplete root formations were excluded during data selection. Finally, 454 objects in 357 anonymized PRs were determined and used in this work. The original images with DCM format had a size of 2943 × 1435 pixels that were initially converted to PNG format in MATLAB. Afterwards, the training, validation, and testing folders were randomly allocated in ratios of 80%, 10%, and 10%, respectively. The training set contained 286 images, while the validation and test sets comprised 36 and 35 images, respectively. There was only one class named lesion. Then, an oral and maxillofacial radiologist (BÇ) with more than 7 years of experience labeled images manually. PyTorch and Google CoLab were primarily used to implement and evaluate models.

### Labeling

Data labeling is an integral part of an AI-based detection application. Specifically, deep learning-based image detection problems require bounding boxes and class names. So, data need to be labeled beforehand. Data labeling, also called data annotation, is a process of adding information, labels, to raw data so that a deep learning model can learn from them. High-quality images, together with accurately annotated data, can provide better solutions. As an open and online annotation tool, Labelme (MIT, USA) was used in this work. The labeling process manually defined the presence of periapical lesions with bounding boxes for each case in each PRs. Any individual areas with periapical lesion was manually chosen by a bounding box. Then, labeled data were delivered to the deep learning models for training, validation, and test steps. In sum, labeling was performed in two phases. Firstly, the data were labeled by an oral and maxillofacial radiologist with more than 7 years of experience. Then, the data were re-investigated and labeled by the same expert in a month, which agreed with the previous step.

### Image pre-processing

Following the preparation and labeling process, raw data were pre-processed before applying deep learning models. Traditional histogram equalization re-maps the image with the assumption that image quality is uniform. When it isn’t distributed uniformly, adaptive histogram equalization is superior to typical histogram equalization. Adaptive histogram equalization maps the image by considering each pixel’s local distribution. When a grayscale distribution is mostly localized, close pixels must be mapped to different grayscales, which is ultimately solved by limiting contrast.

Histogram equalization is calculated with an estimate of the cumulative distribution functions. If M, N and h_i,j_(n) refer the number of pixels, intensity levels and histogram of (i,j) area for *n* = 0, 1, 2,..., N−1, the approximation of the cumulative distribution function is given as follows.

$$f_{i,j}\left(n\right)=\frac{\left(N-1\right)}{M}\sum_{k=0}^{n}h_{i,j}(k)$$

**Figure 2.**
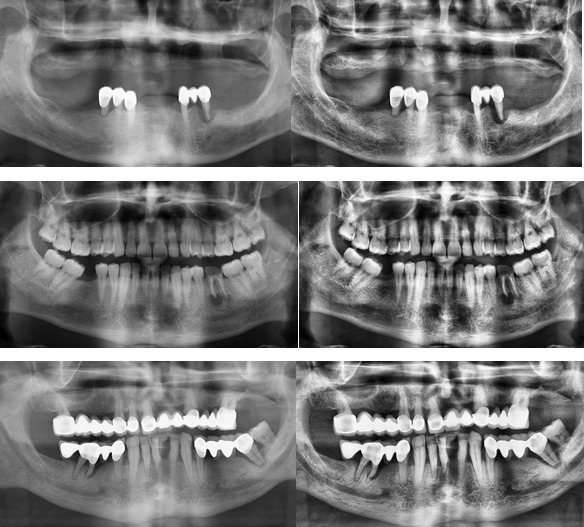
Panoramic image examples with lesions (left) and corresponding pre-processed versions (right).

It can provide a uniform density function. This process is called histogram equalization; region contrast is also increased to its maximum value. Contrast limiting is used to solve this issue by thresholding the slope of f_i,j_(n) to a desired value. Adaptive histogram equalization with 8 × 8 filter and contrast limiting were applied to improve image quality. Figure 2 presents example panoramic images including periapical lesions in row and pre-processed form.

Another pre-processing is resizing. The larger images the longer the training duration it provides. So, all images were resized to a scale of 1333*800 pixels while the aspect ratio is kept constant.

### Object detection with deep learning

Briefly stated, deep learning models perform three main tasks—detection, classification, and segmentation—in computer vision applications. Firstly, object detection aims to differentiate objects in the input image or video. It identifies where objects are and which class they belong to. Deep learning-based object detection models are designed to not only classify the objects present in an image but also provide their precise spatial coordinates (bounding boxes) that outline the object’s location. Secondly, classification task deals with input images with only one object class in it, then determines which class it belongs. Lastly, image segmentation approximates pixel-wise mask of each object in the input image, identifying boundaries and shapes of different objects. In sum, deep learning models learn from a labeled data set during the training phase, which enables models to generalize and perform tasks they are trained for.

Following the data preparation and image pre-processing, data were sent to the deep learning models. Training batch sizes were 16. SGD optimization algorithm is used with learning rate of 0.01, momentum of 0.9 and weight decay of 0.0001. Step learning rate scheduler is used as optimizer. A total of 10 state-of-the-art deep learning models for object detection were implemented. It includes two-stage and one-stage detectors like Faster R-CNN, RetinaNet, SSD, FoveaBox and YOLOv3 with different backbones like ResNet50, ResNet101, DarkNet53, VGG16. Several other models using the same detector but with different integrated mechanisms are also included in this work. The mechanisms are designed to address certain limitations, for instance, dynamic training mechanism for Dynamic R-CNN, a mechanism including a sequence of detectors for Cascade R-CNN, the mechanism with tree main components for Libra R-CNN, a mechanism related to bounding box regression for Side-Aware Boundary Localization (SABL) and anchor-free detection mechanism for Adaptive Training Sample Selection (ATSS).

Faster R-CNN is a well-known two-stage object detection framework.^13^ It uses region proposals instead of selective search algorithm and additionally solved drawbacks of previous versions of it. Convolutional feature map is obtained by supplying input images to convolutional neural network. Separate network predicts region proposals on the feature map, followed by reshaping using region of interest pooling and classifying image in region proposed. RetinaNet is a one-stage object detection framework that proposes focal loss function concept to deal with class imbalance issue in training process.^14^ It is formed by a backbone network and two subnetworks. The backbone computes a convolutional feature map using input image. First subnet takes backbone’s output and performs convolutional object classification, which is followed by second subnet for bounding box regression. YOLOv3 is another one-stage object detection framework with its new backbone compared to its previous version.^15^ The idea of residual connections is used to improve feature extraction and bounding box prediction with three different scales. SSD, Single Shot MultiBox Detector, is a single deep neural network that doesn’t need proposal generation and feature sampling stages.^16^ It combines predictions from feature maps at different resolution, which enables to detect object different sizes.

Libra R-CNN addresses imbalances in training process to improve detection performance.^17^ Sample, feature and objective level imbalances are mitigated by integrating three components, IoU-balanced sampling, balanced feature pyramid, and balanced L1 loss, respectively. Dynamic R-CNN deals with the inconsistency between fixed network settings and dynamic training steps to improve training procedures of detection.^18^ During training process, it regulates intersection over union as label assignment criteria and shape of regression loss function automatically depending on proposals. Cascade R-CNN includes a sequence of detectors which are sequentially trained with higher intersection over union thresholds.^19^ Using output of the former detector as training set for the next both improves quality and minimizes overfitting. FoveaBox is an accurate and flexible and anchor-free detection framework.^20^ Instead of using pre-defined anchors for localization and scaling, FoveaBox utilizes category-sensitive semantic map prediction and category-agnostic bounding box for each location without anchor reference. SABL addresses precision of bounding box regression to improve object detection performance.^21^ Instead of predicting center and size of objects, precise localization is performed by localizing each side of a bounding box with a network branch. Two phase process includes movement prediction to the bucket and pinpointing object precisely in it. Adaptive Training Sample Selection (ATSS) is another anchor-free detection framework focusing on training sample definition that affects the performance and the idea of tiling multiple anchors.^22^ ATSS selects positive and negative input samples depending on objects’ statistical characteristics. Table 1 outlines prominent features of each model briefly.

**Table 1.** The key characteristics of the deep learning models

	Key characteristics briefly
**Faster R-CNN**	It introduced the Region Proposal Network, which efficiently generates region proposals or candidate object bounding boxes for potential object regions.
**RetinaNet**	It introduced a Feature Pyramid Network to extract multiscale feature representations and the Focal Loss, which addresses the class imbalance problem.
**YOLOv3**	It predicts object classes and bounding box coordinates directly in a single pass over the network, resulting in faster inference speed compared to two-stage detectors.
**SSD**	It performs object class and bounding box predictions in one pass, providing in faster execution for real-time applications.
**Libra R-CNN**	It addresses the limitation caused by the imbalance during the training process. It integrates three novel components to reduce the imbalance in three levels.
**Dynamic R-CNN**	It addresses the fixed network settings to improve consistency and performance. It adjusts label assignment criteria and loss function automatically.
**Cascade R-CNN**	It uses a sequence of detectors to address the issues such as overfitting during training and inference-time mismatch.
**FoveaBox**	It is an anchor-free one-stage detection framework to improve performance and generalization ability that are limited to the design of pre-defined anchors.
**SABL**	It addresses unsatisfactory precision of bounding box regression limiting detection performance. It localizes each side of the bounding box to a dedicated network branch.
**ATSS**	It focusses on the performance improvement by automatically selecting positive and negative samples according to statistical characteristics of object.

ATSS, Adaptive Training Sample Selection; SABL, Side-Aware Boundary Localization.

Transfer learning principle is applied in this study. We utilized detectors that had been previously trained on the COCO (Common Objects in Context) data set. The essence of transfer learning lies in harnessing the feature representations learned from pre-existing models. Typically, these pre-trained models have been trained with substantial data volumes that are considered a standard benchmark for applications within the realm of computer vision. This approach allows us to utilize the weights of the pre-existing models as initial parameters for a new application—in this case, periapical lesion detection. Essentially, the transfer of knowledge from previously learned tasks to new tasks can potentially enhance performance significantly.

Model performances were evaluated by mean average precision (mAP), precision, recall, F1 score, accuracy and some other COCO evaluation metrics. Precision-recall curve and area under curve (AUC) were also investigated.

### Evaluation metrics

As the adoption of deep learning algorithms in detection applications continues to expand, the need for reporting effective and comprehensive evaluation criteria has become increasingly important. Evaluation metrics are key to understanding a model’s strengths and weaknesses.

The mAP is a standard metric to evaluate the effectiveness of object detection models in computer vision. It is a common and well-accepted metric for many detection algorithms and benchmark challenges such as Pascal, VOC and COCO challenges. mAP is calculated depending on the Intersection Over Union (IOU).

Object detection tasks are evaluated by mAP, which is a standard metric. Many object detection algorithms and benchmark challenges use it to evaluate models. For instance, detectors like Faster R-CNN, YOLO, SSD, MobileNet, on the other hand Pascal, VOC and COCO challenges utilize mAP.

Calculation of mAP is related to IoU. IoU measures the overlap between the predicted bounding box and the ground truth. As it approaches to 1, it indicates how close the predictions are to the ground truths.

(1) $$IoU=\;\frac{area\left(ground\;truth\;\cap predicted\right)}{area\left(ground\;truth\;\cup predicted\right)}$$

IoU is a key component in the construction of a confusion matrix, a tabulation of predicted and actual classifications for each class. This matrix provides an overview of the classifier’s performance by identifying true positives, true negatives, false positives, and false negatives. Accuracy, precision, recall and F1-score are calculated by confusion matrix. Table 2 presents the metrics and how they are calculated based on confusion matric components.

**Table 2.** Evaluation metrics and their calculations based on confusion matrix

Precision	$\frac{True\;Positive}{True\;Positive+False\;Positive}$
Recall (sensitivity)	$\frac{True\;Positive}{True\;Positive+False\;Negative}$
Accuracy	$\frac{True\;Positive+True\;Negative}{All\;predictions}$
F1-Score	$\frac{2*True\;Positive}{2*True\;Positive+False\;Positive+False\;Negative}$
Average precision	$AP_{threshold}=\int_{0}^{1}p\left(x\right)dx$
Mean average precision for n-classes	$mAP_{threshold}=\frac{1}{n}\sum_{i=1}^{n}AP_{i}$

- Accuracy: This is the proportion of correct predictions (both positive and negative) in relation to the total number of predictions.
- Precision: Also known as the positive-predictive value, it’s the proportion of correct positive predictions in relation to the total number of positive predictions.
- Recall: Also known as sensitivity, true-positive rate, it’s the proportion of correct positive predictions in relation to the total number of actual positives.
- F1-score: This is the harmonic mean of precision and recall, and it tries to find the balance between these two metrics.

In object detection tasks, IoU plays a crucial role in determining precision. If the IoU of a detected object with the corresponding ground truth exceeds a specific IoU threshold, typically 0.5, it’s classified as a true positive. mAP signifies the mean of the average precision (AP) for each individual class. AP is determined by calculating the area under the precision-recall curve, taking into account different IoU thresholds.

The precision-recall curve is a graphical representation used to evaluate a model’s classifier quality. It illustrates the tradeoff between precision and recall at varying thresholds. One way to summarize the precision-recall curve is to calculate the area under this curve (AUC). The larger the area, the better the balance between precision and recall, and hence the better accuracy the model has. The COCO metrics are illustrated within the precision-recall curve using the AUC. In simple terms, C75 and C50 refer AUC for IOU of 0.75 and 0.5, respectively. Loc stands for AUC ignoring localization errors. Sim, Oth and FN demonstrate AUC while removing super-category class confusions, class confusions and all remaining errors, respectively.

### Benchmarking

Performance comparison is a valuable step to determine the most suitable solutions for a specific task. In addition to developing and implementing deep learning models, comparisons with external data and human observer were generally needed. Based on the need for external validation to ensure a more comprehensive and robust assessment of our deep learning models' performance, we performed comparisons with an external data and human observers.

#### Comparison with external data set

After the training of the deep learning models was completed, they were tested with an external public data set in addition to the existing test data. Reporting models' performances on external data set contribute evaluations in terms of generalization and robustness. The Tufts Dental Database is used as an external public data set.^23^ It classifies 1000 PRs into five different levels such as anatomical location, peripheral characteristics, radiodensity, effects on the surrounding structure, and the abnormality category. As they are not specifically collected for the presence of periapical lesion, we have only determined 41 PRs for the present work.

#### Comparison with human observers

Models' performances were also compared with human observers. An endodontist (ES) and a radiologist (BÇ) who have experiences for more than 7 years’ experience evaluated the same external data set. The radiologist’s evaluation was isolated by a washout period of 6 weeks. Experts were separately asked to label the images in the external data set using the labeling tool in a room without patients and/or students. The time duration was also noted. Their observations were compared with the ground truth to calculate the evaluation metrics which are also used for evaluation of deep learning models.

## Results

Training process was followed by testing procedures. Performance metrics were calculated for each model, which was given in Table 3. Detection performances were between 0.83 and 0.95 while accuracy was between 0.67 and 0.81. F1 score which is a combination of precision and recall, called harmonic mean, is between 0.8 and 0.89. The best detection performance was obtained by RetinaNet and ATSS in addition to the highest accuracies together with YOLOv3. ATSS provided the highest F1 score of 0.89.

**Table 3.** Performance results for each model

Models	Backbone	mAP1	Accuracy	Precision	Recall	F1 Score	Training duration
Faster R-CNN	ResNet-101	0.926	0.747	0.910	0.788	0.840	2 h 16m
RetinaNet	Resnet-101	0.953	0.777	0.910	0.743	0.814	1 h 42m
YOLOv3	DarkNet53	0.845	0.772	0.829	0.918	0.871	56m
SSD	VGG16	0.832	0.673	0.825	0.785	0.8	1 h 14m
Libra RCNN	Xception-101	0.920	0.740	0.890	0.784	0.833	2 h 34m
Dynamic R-CNN	Resnet-50	0.904	0.747	0.930	0.780	0.848	1 h 36m
Cascade R-CNN	Resnet-101 FPN	0.913	0.724	0.897	0.790	0.840	1 h 54m
FoveaBox	ResNet-50 FPN	0.895	0.745	0.931	0.788	0.853	1 h 5m
SABL Faster R-CNN	ResNet-101 FPN	0.894	0.732	0.921	0.780	0.844	2 h 23m
ATSS	ResNet-101 FPN	0.939	0.812	0.886	0.906	0.895	1 h 33m

ATSS, Adaptive Training Sample Selection; SABL, Side-Aware Boundary Localization.

**Figure 3.**
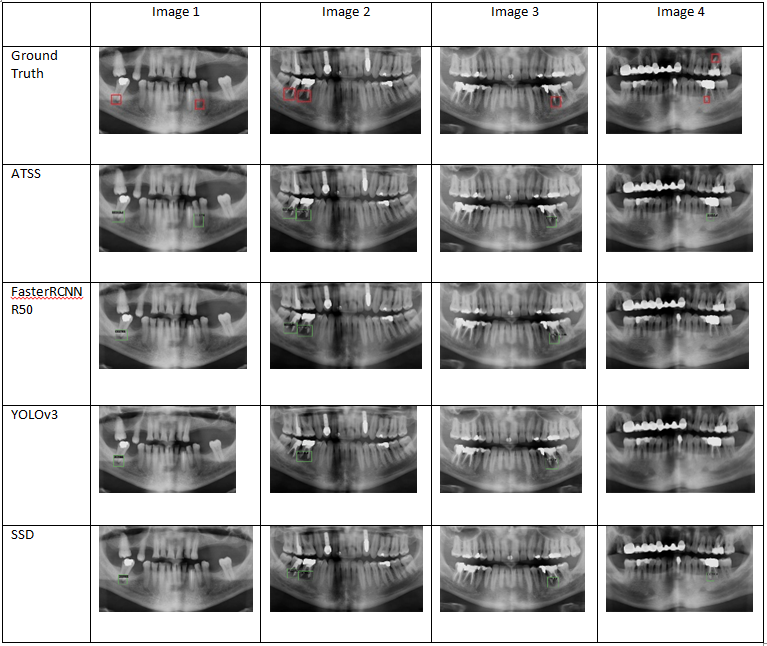
Output images with predicted bounding box for localization, class label and confidence level.

**Figure 4.**
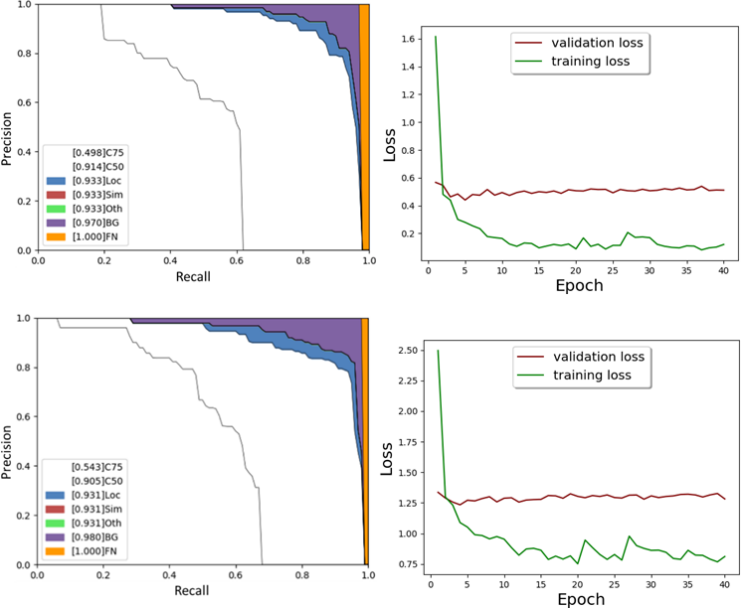
Precision recall and loss curve for RetinaNet and ATSS. ATSS, Adaptive Training Sample Selection.

Example outputs that contained bounding box prediction, class name and confidence were presented in Figure 3. Ten different deep learning-based detectors’ detection performance varies between 0.832 and 0.953. From highest performance to lowest performance—ATSS, Faster RCNN, YOLOv3 and SSD—four models are chosen, their outputs are presented with four images together with the ground truth. For presentation and visualization purposes, images in Figure 3 were given after cutting the image to focus the area of interest. It is seen that periapical lesions are mostly detected successfully, however, one of the periapical lesion in Figure 4 cannot be detected.

Furthermore, ATSS and FoveaBox outputs were analyzed using precision-recall curves as the best performing models. Both models had ResNet-101 as a backbone. Their precision-recall curves and loss curves were presented in Figure 4.

AUC from precision-recall curve using detection evaluation metrics used by COCO indicated that AUC would be 0.933 and 0.931 for RetinaNet and ATSS, respectively. There wasn’t any error related to super-category false-positives and class confusions. When background confusions were removed, AUC would be 0.97 and 0.98, respectively. Overall, errors were originated by location and background errors.

Ideally, multicenter data can help mitigate overfitting and enhance the generalizability of the models. Regarding that, the trained models were further tested with an external data set collected from a different device. Table 4 below presents the results.

**Table 4.** Performance evaluations with an external data set

Testing with the Tufts Dental Database						
Model	Backbone	mAP1	Accuracy	Precision	Recall	F1 Score
Faster R-CNN	ResNet-101	0.838	0.642	0.9	0.692	0.782
RetinaNet	Resnet-101	0.910	0.642	0.818	0.75	0.782
YOLOv3	DarkNet53	0.757	0.636	0.70	0.875	0.77
SSD	VGG16	0.777	0.583	0.7	0.777	0.736
Libra RCNN	Xception-101	0.780	0.571	0.8	0.666	0.727
Dynamic R-CNN	Resnet-50	0.852	0.75	0.9	0.818	0.857
Cascade R-CNN	Resnet-101 FPN	0.871	0.571	0.8	0.66	0.723
FoveaBox	ResNet-50 FPN	0.726	0.583	0.7	0.777	0.736
SABL Faster R-CNN	ResNet-101 FPN	0.797	0.692	0.9	0.75	0.818
ATSS	ResNet-101 FPN	0.787	0.615	0.8	0.727	0.761

ATSS, Adaptive Training Sample Selection; SABL, Side-Aware Boundary Localization.

Regarding the performances of the models, mAP1 varied between 0.72 and 0.91 while the accuracy was between 0.57 and 0.75. The F1 score which is related to both precision and recall was between 0.72 and 0.85. The RetinaNet yielded again the best detection performance with mAP of 0.91.

We also compared model performances with human observers. Table 5 presents the performance of human observers.

**Table 5.** Expert observations on the same external data set

	mAP50	Accuracy	Precision	Recall	F1-score	Time	Time for each image
The Endodontist	0.97	0.979	1	0.97	0.98	28 min	41 s
The Radiologist	1	1	1	1	1	24 min	35 s

The endodontist successfully labelled periapical lesions except only one (47 out of 48) while the radiologist detected all (48 out of 48). When it is compared to the predictions of the deep learning models, experts provided better performances. However, deep learning models are superior in terms of execution time, *i.e*. their predictions took only a second.

## Discussion

Deep learning-based object detection frameworks can be categorized into two different classes such as region proposal-based techniques and regression/classification-based techniques. Region proposal-based techniques create region proposals and then classify each of them into pre-defined classes. For instance, R-CNN, Fast R-CNN, and Faster R-CNN are well-known region proposal-based models. Regression/classification-based techniques predict object locations directly through one stage. As an example, YOLO, SSD, and RetinaNet are regression-based methods.

PRs are one of the most frequently used imaging types in daily routines of dental clinics. They are comparatively safer and scan wider oral structures. However, considering complicated dental cases, time limitations, burden of the clinicians, a digital tool is now a need for clinicians to improve dental healthcare and overall quality. To achieve that, we applied the state-of-the-art deep learning detection models to pre-processed panoramic dental radiographs for periapical lesion detection. Image pre-processing step improved image quality and homogeneity. On the other hand, direct comparison of the results to previous works weren’t always possible. Prior works used different types of radiographies such as cone beam CT and periapical images, different purposes like segmentation frameworks and various evaluation metrics.

Zheng et al used dental cone beam CT images from 20 patients to do lesion detection using constrained dense U-Net with oral-anatomical knowledge integration.^24^ Precision and recall for root lesion detection for dense U-Net with cross-entropy loss and focal loss were respectively 0.65–0.7 and 0.68–0.8. Anatomically constrained dense U-Net provided precision and recall as 0.9 and 0.84. Endres et al compared assessments of 24 oral and maxillofacial surgeons with predictions of a deep learning model on PRs.^25^ U-Net model architecture with 28 layers was used. Deep learning model provided average precision of 0.6 and F1 score of 0.58. Orhan et al used 3D cone beam CT scans with U-Net architecture to segment 153 periapical lesions from 109 patients.^26^ It was reported that 142 objects out of 153 were correctly detected, giving reliability of 0.92. Ekert et al presented deep convolutional neural networks with seven layers to detect apical lesions on PRs.^27^ 85 PRs were used. AUC, recall, specificity were 0.85, 0.65 and 0.87, respectively. Krois et al trained U-Net type deep learning models to segment apical lesions on PRs using 650 PRs from each of two centers.^28^ F1 score was obtained between 0.32 and 0.54 for different cases. Bayrakdar et al applied U-Net based deep convolutional neural network to segment apical lesions using 470 anonymized dental PRs.^29^ Sensitivity, precision and F1 score were 0.92, 0.84, and 0.88, respectively. Li et al detected apical lesions using convolutional neural networks on 476 periapical images, 65 of which had lesions.^30^ Accuracy, sensitivity, specificity, precision and recall were 0.92, 0.94, 0.9, 0.92 and 0.94, respectively. Pauwels et al utilized convolutional neural networks to detect periapical lesions with and without various filtering settings.^31^ The performance of the AI-based approach was then compared to that of human observers. The sensitivity, specificity, and ROC-AUC varied between 0.79 and 1, 0.88 and 1, 0.86 and 1, respectively, for three different cases while human observers provided 0.58, 0.83 and 0.75 for sensitivity, specificity, and ROC-AUC, respectively. Simulated periapical lesions on periapical radiographs were used to overcome the possible mistakes by clinicians during the labeling process, which allowed for perfect annotation of training and validation data without the need for human data annotation. Moidu et al applied convolutional neural network, YOLOv3, for periapical lesion scoring based on the periapical index scoring approach with five classes defining the case from normal to a severe situation.^32^ Score 1, normal periapical structure, provided 90% true prediction while true prediction rate of the other four scores varied between 30 and 71%. When the categorization was transformed into two classes as healthy and diseased, the true prediction was obtained as 76.6 and 92% for each class, respectively. 92.1% sensitivity/recall, 76% specifcity, 86.4% positive-predictive value/precision, and the accuracy, F1 score were 86.3%, 0.89, respectively.

Table 6 comprehensively compared previous works and this work in terms of the task performed, the type of image used, the model used, the data size—the number of image and the evaluation metrics reported.

**Table 6.** Comparison of the results with previous works

Author	Task	Type of Image	Model	Data size	Metrics
Zheng et al	Segmentation	CBCT	U-Net	20	Precision: 0.65–0.9 Recall: 0.68–0.84
Endres et al	Segmentation	Panoramic	U-Net	2902	Precision: 0.6 F1 score: 0.58
Orhan et al	Segmentation	CBCT	U-Net	153	Estimated precision: 0.95 Estimated Recall: 0.89 Estimated F-measure: 0.93
Ekert et al	Segmentation	Panoramic	CNN	85	AUC: 0.85 Recall: 0.65 Specificity: 0.87
Krois et al	Segmentation	Panoramic	U-Net	650	F1 score: 0.32–0.54
Bayrakdar et al	Segmentation	Panoramic	U-Net	470	Precision: 0.84 Sensitivity: 0.92 F1 score: 0.88
Li et al	Detection	Periapical	CNN	476	Accuracy: 0.92 Sensitivity: 0.94 Specificity: 0.9 Precision: 0.92 Recall: 0.94
Pauwels et al	Classification	Intraoral	CNN	280	Sensitivity: 0.79–1 Specificity: 0.88–1 ROC-AUC: 0.86–1
Moidu et al	Detection	Periapical	YOLOv3	1950	True prediction: 30–90% Precision: 0.86 Sensitivity/recall: 0.92 Specificity: 0.76
This work	Detection	Panoramic	Faster R-CNN RetinaNet YOLOv3 SSD Libra RCNN Dynamic R-CNN Cascade R-CNN FoveaBox SABL Faster R-CNN ATSS	357	mAP: 0.83–0.95 Accuracy: 0.67–0.81 Precision: 0.82–0.93 Recall: 0.74–0.91 F1 score: 0.8–0.89

AUC, area under the curve; CBCT, cone beam CT; CNN, convolutional neural network; ROC, receiver operating characteristic.

ATSS, Adaptive Training Sample Selection; SABL, Side-Aware Boundary Localization.

Previous works related to presence of periapical lesion were comprehensively examined. However, it was seen that direct comparison was not always possible due to differences in the task performed, the type of image used, the type of method reported, the number of images used in the data set and lastly the evaluation metrics reported. Regarding the comparison of previous works with this work, each metric was focused. Detection performance is generally evaluated by mAP metric, which is a standard metric for computer vision-based challenges and data sets, like Pascal, VOC and COCO, etc. Neither of the previous works was reported mAP. The other metric is accuracy. Accuracy was only reported by Li et al, however, they used different types of imaging, intraoral periapical image, in their work, which should be taken into account while comparing the studies.^30^ Precision, recall and F1 score obtained via 10 different models showed similar or mostly superior performance compared to previous works. As a same deep learning model of a previous work, YOLOv3 was also used by Moidu et al but using intraoral periapical radiographs, a different type of imaging modality for detection purposes.^32^ Based on the metrics they reported, it was not possible to compare detection performance. This work showed better performance for their first scenario with 5-score case. The performance was similar for their second scenario with 2-score case, healthy and diseased.

On the other hand, two other work focused on the possible role of the AI for lesion detection compared to human observers. Hamdan et al showed that dentists’ performance for localization and detection of apical radiolucencies were improved during computer-aided session.^33^ Specificity and sensitivity were improved 23.1 and 8.2% after the AI-aided session. It provided very limited results to compare this work, but sensitivity and specificity values were similar. Zadrozny et al investigated the reliability of the automatic assessment of some pathologies, including periapical lesion, on PRs generated by AI and three dentists with different years of experiences.^34^ Interclass correlation coefficient (ICC) for three evaluators proved the good credibility of the ground truth (ICC >0.75). Compared to ground truth, statistical evaluation of AI-based solution provided acceptable reliability for missing teeth, prosthetic restorations, dental fillings, endodontically treated teeth, residual roots and periodontal bone loss (sensitivity between 0.8 and 0.9) while evaluation of caries (ICC = 0.681) and periapical lesions (ICC = 0.619) provided adequate reliability. No other metrics were reported for comparison purposes.

The present work performed testing with external data, which shows how close and successful models are in generalizing periapical lesion detection. Considering that the device/scanner and exposure parameters used for the Tufts dental database are different than ours, our testing results with an external data set show a consistent trend with our previous results. If a periapical lesion specific database collected from different devices were used during the training process, the results would have been higher than the existing case. On the other hand, comparison with human observers may give us a more realistic benchmark of our models' effectiveness in a real-life scenario. The experts provided superior performance than AI models. This is as expected considering their professional experiences, limited testing size and limited variety and number of observers. It might be different with clinicians at different levels such as students and new graduates. Additionally, experts identified periapical lesions in agreement with each other (47 *vs* 48 correctly detected periapical lesions out of 48 periapical lesions). A reliable decision-support system might be a powerful tool to help clinicians with almost instant predictions in their busy work schedules. Next, investigating the effect of AI in decision-making among a wide range of observers with a proper technical infrastructure integrating into the clinic with embedded automatic graphical and assessment tools can contribute to the existing literature.

While deep learning has significantly advanced the field of detection applications, several challenges and limitations remain before integrating AI applications into the clinics. Ideally, studies should be carried out with very large, balanced, and high-quality image data sets from different devices of multicenters. There is a lack of reliable, large, public, and independent data sets to use and compare results. The field of research regarding the applications of deep learning models is robust and expansive in dentistry. A significant issue is the lack of standardization and comparability across different studies. The metrics employed to evaluate the performance and accuracy of these models vary widely from one study to another. These can range from precision, recall, accuracy, F1 score, AUC, among others. While each of these metrics has its own merits and is suitable for certain situations, the inconsistent use across different studies impedes the direct comparison of results. Without a uniform standard for evaluating and presenting results, it becomes challenging to compare the effectiveness of different models or to draw conclusions about best practices and superior strategies. Several guidance documents are useful for planning, conducting and reporting of AI studies for authors, reviewers and readers in dental research.^35^

## Conclusion

This work applied 10 different deep learning models for periapical lesion detection. It also compared their performances with external data set, experts’ observations, and previous works. It was concluded that deep learning models can successfully detect periapical lesions on PRs. Current limitations should also be addressed in the future for clinical integration.
